# Efficacy and safety of low-dose X-rays based intraoperative radiotherapy for high-grade gliomas: a single-center retrospective study

**DOI:** 10.3389/fonc.2025.1548276

**Published:** 2025-05-05

**Authors:** Zhongcheng Wen, Jun Liu, Yang Bai, Minghao Zhou, Jiajie He, Ying Xu, Ying Yan, Fuyong Li, Shun Gong, Jianan Li, Peng Cao

**Affiliations:** ^1^ Department of Neurosurgery, General Hospital of Northern Theater Command, Postgraduate Training Base of Dalian Medical University, Shenyang, China; ^2^ Department of Neurosurgery, General Hospital of Northern Theater Command, Postgraduate Training Base of Jinzhou Medical University, Shenyang, China; ^3^ Department of Radiotherapy, General Hospital of Northern Theater Command, Shenyang, China; ^4^ Department of Neurosurgery, People’s Hospital of Liaoning Province, People’s Hospital of China Medical University, Shenyang, China

**Keywords:** high-grade gliomas, intraoperative radiotherapy, surgery, survival, efficacy, safety

## Abstract

**Background:**

Intraoperative radiotherapy (IORT) is an emerging local therapy in the surgery of intra-axial brain tumors to improve clinical outcomes and accelerate the adjuvant oncologic therapy. Despite its use in neuro-oncology, the data regarding the role of IORT in the treatment of high-grade gliomas (HGGs) is sparse. Here we reported our single-institutional evidence concerning the efficacy and safety of IORT in the management of HGGs.

**Methods:**

A total of thirty patients diagnosed with HGGs who underwent surgical treatment and IORT at our center between October 2021 and October 2023 were included. Clinical data were collected and analyzed, including surgical parameters such as the gross total resection (GTR) rate, follow-up assessments of treatment responses (Karnofsky Performance Status [KPS] scores), treatment-related complications, overall survival (OS), and subsequent therapeutic interventions. Multivariable Cox regression analyses were performed to identify independent risk factors for survival in patients with HGGs.

**Results:**

The median IORT dose was 12 Gy prescribed to the applicator surface using the INTRABEAM system. The median OS was 11.0 months (IQR: 7.8–14.3), with a 1-year survival rate of 46.7%. No severe radiation-related adverse events, such as cerebral radiation necrosis or wound-related complications, were recorded. Kaplan-Meier analyses showed that patients who received post-operative radiotherapy and chemotherapy after IORT had better clinical outcomes than those who did not. Multivariable regression analyses indicated post-operative radiotherapy was independently correlated with favorable clinical outcomes.

**Conclusion:**

Low-dose X-rays based IORT at doses of 10-12 Gy is generally safe for HGGs. Future prospective large-scale studies are needed to further evaluate the efficacy and safety of IORT with escalating doses. Even with the use of IORT, post-operative radiotherapy is essential for improving clinical outcomes of HGGs. This study provides clinical data on IORT for HGGs, which may represent a promising therapeutic approach for managing this disease.

## Introduction

Gliomas are classified into grades based on their histological features and molecular characteristics. Grade I gliomas are considered benign, while grades II, III, and IV are malignant. Grade IV glioblastomas (GB) are the most common malignant gliomas, accounting for approximately 50% of all cases, which are most frequently diagnosed in individuals over the age of 65 (1). High-grade gliomas (HGGs), including grade III-IV astrocytomas and GB, represent the most prevalent malignant primary brain tumors in adults. Despite their relative rarity, HGGs are characterized by aggressive clinical behaviors and poor prognoses, making them one of the most challenging diseases in modern neurosurgery. The standard of care, known as the Stupp protocol, involves maximal safe resection followed by radiotherapy (60 Gy in 30 fractions) and chemotherapy (concurrent and adjuvant temozolomide) (2). Despite multimodal therapeutic regimens, HGG patients still have a high risk of early relapse and a high mortality rate. Nonetheless, significant progress has been made over the past decades in the pursuit of improving overall survival.

Given that the majority of tumor recurrences occur in or near the resection cavity, many novel therapies focus on combating these local recurrences by implementing treatments directly within the tumor bed (3). Among those, intraoperative radiotherapy (IORT) has emerged as a promising treatment option for HGGs. IORT delivers precise doses of radiation, including high-dose electrons and low-dose X-rays, to the tumor bed immediately following resection. This approach theoretically targets or destroys residual microscopic tumor cells at the resection cavity margin, which are impossible to be identified (3, 4). For decades, photon-based IORT has shown promise in controlling various cancers, including head and abdominal tumors (5–8). Recently, the INTRABEAM system (Carl Zeiss Meditec AG, Oberkochen, Germany), a mobile IORT unit, has been introduced to deliver low-energy X-rays (50 kV) with rapid dose attenuation^9.^ This system has shown potential in minimizing radiation exposure to adjacent tissues. Recent studies have highlighted its feasibility in recurrent GB management, with cerebral radiation necrosis (CRN) rates reported to be below 5% (2). A series of studies have shown that patients with GB, ependymoma, and brain metastases may benefit from additional low-dose IORT provided by this system (9–13). Emerging evidence suggests that low-dose IORT may improve therapeutic outcomes in patients with recurrent isocitrate dehydrogenase wild-type (IDHwt) GB (2). Previous studies have demonstrated the safety of IORT and its potential to extend survival, although clinical samples have been limited. So far, clinical data concerning the feasibility of IORT for the treatment of HGGs remain sparse. Our study further increases the clinical sample size and analyzes the factors influencing survival, aiming to provide more clinical data on IORT.

## Materials and methods

### Patient collection

This study was performed in accordance with the Declaration of Helsinki. It was approved by the local institutional review board (Y2022-100) and registered with Chinese Clinical Trial Registry website (ChiCTR2200063349). A retrospective review was conducted on all patients with gliomas who received surgical treatment at General Hospital of Northern Theater Command between October 2021 and October 2023.

The inclusion criteria for the participants were: (1) Adult patients (>18 years old) diagnosed with HGGs that were confirmed by postoperative pathologic and immunohistochemistry analysis; (2) Preoperative KPS ≥ 50; (3) Patients who signed informed consent to receive IORT during treatment. Exclusion criteria were: (1) Patients under 18 years of age (this study specifically focused on adult patients); (2) Presence of other severe illnesses (such as coronary artery disease, renal failure, etc.); (3) Pregnant or breastfeeding women; (4) Patients with multiple lesions or distant metastasis. In total, 30 cases of HGGs were included in the cohort.

Clinical data, including demographics (age and sex) and medical history, were collected. All patients received a physical examination and KPS evaluation. Tumor location and size were evaluated by gadolinium-enhanced magnetic resonance (MR) imaging. Gliomas located within 1 cm of functional areas, including sensorimotor areas, language areas, basal ganglia and internal capsule, thalamus, and visual cortex, were defined as functional area gliomas. Detailed information on all patients with HGGs is shown in **Table 1**.

**Table 1:** Clinical characteristics, treatment process, and outcomes of each patients included.

Pat. #	Sex & Age	Location	Functional areas involved	Tumor volume (cm^3^)	Recurrent tumor	Pre-op KPS (%)	EOR	Pathology	WHO grading	Ki-67	LOS	Complications	Post-op KPS at 6 mo (%)	Postoperative therapy	FU (month)	OS (month)
1	67,M	L, F	MA	25.6		80	GTR	A	4	15	28	Muscle weakness	70	EBRT	37	10
2	64,M	R, P	MA, SA	15.0		80	STR	A	3	40	17		70	Chemo	36	12
3	77,M	L, F	MA, LA	67.2		80	GTR	GB	4	50	13	Dysphasia	60		35	9
4	39,W	R, F		9.8	+	80	GTR	A	4	50	21	Headache	60		28	8
5	21,M	L, FP		41.9		90	STR	A	3-4	20	17		70	EBRT	28	16
6	32,M	R, PO	VA	125.6		90	GTR	A	4	60	25	Headache	90	EBRT+Chemo	28	23
7	70,W	L, F	LA	67.7		70	GTR	A	3-4	80	29				27	4
8	79,W	L, F	MA	22.9		80	GTR	GB	4	50	15	Headache, Seizure	40	EBRT+Chemo	27	15
9	40,W	L, F		74.3		80	GTR	A	3-4	60	15		70	EBRT+Chemo	27	16
10	62,M	L, TP		111.0	+	80	GTR	A	3-4	30	13	Headache	70	EBRT	26	13
11	58,M	R, P	MA	17.5		60	GTR	A	4	60	14	Dysesthesia	50		26	13
12	67,W	R, PO	MA	107.0		80	GTR	A	3	30	15		70		26	15
13	60,W	R, FP	MA	14.2		80	GTR	A	3	10	16	Headache	60	EBRT	25	16
14	47,M	L, F		7.9	+	80	GTR	GB	4	70	27		70	EBRT+Chemo	23	11
15	54, M	R, Tha	Tha	88.6		90	GTR	A	3-4	50	17		50		22	10
16	69,W	L, T		44.0		70	GTR	A	4	85	16	Headache, Seizure	40	EBRT	21	8
17	67,M	R, T	MTL	28.0		80	GTR	A	3-4	30	14				20	5
18	66,W	R, F		15.5		90	GTR	A	3	65	27		90	EBRT+Chemo	19	18
19	61,W	R, OP		53.1		80	GTR	A	3	70	11		50		18	8
20	45,M	L, LV	MA	148.0	+	80	GTR	OA	3	35	20	Muscle weakness	50		18	7
21	66,W	R, F	CC, BG	18.5		90	STR	A	3-4	80	26				17	3
22	65,M	L, FT	LA	5.0		80	GTR	A	3-4	90	13	Dysesthesia	80		16	13
23	54,M	R, FT	MA	313.2		90	STR	A	3-4	35	10	Headache			16	4
24	62,W	R, P	SA	24.9		90	GTR	A	3-4	40	16		50	EBRT+Chemo	16	7
25	72,M	R, F	MA	53.5		70	GTR	A	3-4	40	13	Seizure	50	Chemo	15	14
26	29,W	L, F	MA	58.7		90	GTR	A	3-4	40	34	Muscle weakness	90	EBRT+Chemo	14	13
27	76,W	L, PTO		68.0		90	STR	GB	4	80	21	Headache			14	3
28	74,W	R, F	LA	40.3		80	GTR	A	4	80	20	Headache, Dysphasia	80	Chemo	13	12
29	53,M	L, F	MA, LA	31.0		80	GTR	A	3-4	70	14		70		13	11
30	59,M	R, T	BG, Tha	59.0		90	GTR	A	3-4	70	13	Headache	40		13	8

A, Astrocytoma; BG, ganglia; CC, corpus callosum; Chemo, chemotherapy; EBRT, external beam radiotherapy; EOR, extent of resection; F, female; F, frontal lobe; FP, frontoparietal lobe; FT, frontotemporal lobe; FU, follow-up; GB, glioblastoma; GTR, gross total resection; KPS, Karnofsky Performance Scale; L, left; LA, language area; LOS, length of stay; LV, lateral ventricle; M, male; MA, motor area; MTL, medial temporal lobe; O, occipital lobe; OA, oligoastrocytoma; OT, occipitaltemporal lobe; P, parietal lobe; PO, parietooccipital lobe; PTO, parietotemporo-occipital lobe; R, right; STR, subtotal resection; T, temporal lobe; T, thalamus; TP, temporoparietal lobe; VA, visual area.

### IORT procedure

Multimodal techniques, including intraoperative navigation, fluorescein-guided surgery, and electrophysiological monitoring, were utilized to ensure safe, accurate, maximum tumor resection. IORT (Carl Zeiss Meditec AG, Oberkochen, Germany) was performed after tumor resection and subsequent confirmation of HGGs by intraoperative frozen pathological analysis. Important tissues near the radiated areas, including cerebral vessels and the brainstem, were covered with a gelatin sponge to reduce radiation damage. A proper spherical applicator, ranging from 1.5 to 5.0 cm, was selected based on the tumor cavity geometry and adjacent functional brain areas. According to the “tightest fit rule”, the applicator should provide structural support to the tumor margin with mild pressure, which guarantees maximum killing effect against glioma cells in adjacent brain tissues and prevented bleeding and transudation. Next, IORT was administered at dose levels ranging from 10 to 12 Gy. The prescribed dose was directed to the surgical margin at a 2-mm depth (the surface of the applicator). The selection of the 10-12 Gy dose range was based on previous safety data, which demonstrated minimal vascular toxicity and a low risk of CRN (1, 13). Pathological analysis indicates that the toxic infiltration depth is between 2 and 5 mm, which is sufficient for targeting the residual tumor while sparing critical structures (14). After radiation, the applicator was removed from the tumor, and the surgery proceeded as usual without any additional specific requirements.

### Postoperative management

Postoperatively, all patients received contrast-enhanced MRI within one week to assess the extent of resection by comparing preoperative and postoperative T1-enhanced sequences. The diagnosis of HGGs was confirmed via pathological examination. Adverse events, such as radiation encephalopathy, wound-related complications, intracranial infection, cerebral bleeding or ischemia, and nervous system disorders, were monitored during hospitalization and throughout the follow-up period. A personalized treatment plan, including radiotherapy, chemotherapy, and targeted therapy, was formulated based on the patient’s physical condition,economic status (as some patients were unable to afford the costs of subsequent therapies) and personal preferences.

### Follow-up

After surgery, patients were followed up for two years or until death. During the follow-up period, treatment response, treatment-related complications (including both immediate and delayed complications), and subsequent therapies were recorded. Immediate complications occurring postoperatively included dysphasia, muscle weakness, dysesthesia, headache, hydrocephalus, cerebrospinal fluid leakage, meningitis, seizures, postoperative hemorrhage, and cerebral radiation necrosis. Delayed complications included wound infection, scalp edema, and wound dehiscence. Each follow-up included a review of recent medical history, wound examination, and clinical assessment. For surveillance imaging, all patients were required to receive enhanced brain MRI at 1, 3, and 6 months after surgery, with subsequent imaging performed every 6 months thereafter. Overall survival (OS) was defined as the interval from tumor resection to death from any cause.

### Statistical analysis

Data were expressed as percentages (%) for enumeration data or medians [interquartile ranges (IQRs)] for quantitative data. Cumulative survival curves were plotted using the Kaplan-Meier method along with the log-rank test. Cox regression analyses were performed to identify independent predictors for the primary outcome in this cohort. All statistical analyses were conducted using SPSS software (version 22.0, IBM Corp, NY, USA). p < 0.05 were considered statistically significant.

## Results

### Patient characteristics

A total of thirty hospitalized patients with HGGs were treated with IORT. Detailed information on demographics, clinical and radiographic features, and treatment is shown in **Tables 1**, **2**. The median age was 62 years (IQR: 53.3–67.0), among whom 53.3% were male. Thirteen patients (43.3%) had gliomas located in the frontal lobe, and twenty patients (66.6%) had gliomas within functional areas, with motor areas most frequently involved (12 cases).

**Table 2:** Clinical characteristics of all patients with HGGs receiving IORT.

Clinical characteristics	Median (interquartile range), or n (%)
Gender	
Male	16 (53.33%)
Female	14 (46.67%)
Age, y	62.0 (53.3-67.0)
Side	
Left	14 (46.67%)
Right	16 (53.33%)
Functional areas involved	21 (70.00%)
Tumor volume (cm^3^)	43.0 (20.0-68.0)
Recurrent tumor	4 (13.33%)
Pre-operative KPS (%)	80 (80-90)
Extent of resection	
Gross total resection	25 (83.33%)
Subtotal resection	5 (16.67%)
Pathology	
Glioblastoma	4 (33.33%)
Astrocytoma	25 (83.33%)
Oligodendroglioma	1 (3.33%)
WHO grading	
III	6 (20.00%)
III-IV	14 (46.67%)
IV	10 (33.33%)
Ki-67	50.0 (39.0-73.0)
LOS	16 (14-21)
Post-operative KPS at 6 mo (%)	70 (50-75)
Post-operative therapy	
EBRT+chemotherapy	7 (23.33%)
EBRT	5 (16.67%)
Chemotherapy	3 (10.00%)
None	15 (50.00%)
Follow-up time (mo)	21.5 (16.0-27.0)
Survival time (mo)	11.0 (7.8-14.3)

KPS, Karnofsky performance scale; LOS, length of stay; EBRT, external beam radiotherapy.

Of the patients, four (13.3%) with recurrent HGGs received their first tumor resection 1.5, 3.0, 8.0, or 14.0 years ago, and only two of them received standard adjuvant therapy according to the Stupp protocol. Prior to surgery, the median KPS score was 80 (IQR: 75–80).

### Treatment outcomes and adverse events

In this cohort, twenty-five patients underwent total resection, while five patients received subtotal resection, three of whom had gliomas located in functional areas. Among the thirty patients, twenty-five patients had astrocytomas, four had GB and one had oligoastrocytoma. Regarding WHO grading, six patients had WHO grade III gliomas, fourteen had WHO III-IV gliomas, and ten had WHO IV gliomas. The median Ki-67 labeling index was 50.0 (IQR: 39.0–73.0).

The median follow-up was 21.5 months (IQR:16.0–27.0). During the follow-up period, three patients survived. The median OS was 11.0 months (IQR: 7.8–14.3), and the OS rate estimated at one year after IORT was 46.7%. At six months after IORT, the patients had a median KPS score of 70 (IQR: 50–75).

For immediate complications, two patients experienced dysphasia, three patients had muscle weakness, two patients presented with dysesthesia, and ten patients reported headaches. Additionally, three patients experienced seizures postoperatively. No patients experienced delayed complications such as wound infection, scalp hydrops, or wound dehiscence. It is noteworthy that the tumors in these patients were located in the corresponding functional areas of the brain. Therefore, we have no evidence to suggest that these complications were caused by IORT. Furthermore, the incidence rates of headaches and seizures were similar to those observed in conventional craniotomy procedures without IORT.

After surgery, ten patients complained of headache, and seven reported aggravated or new-onset dysphasia, muscle weakness or dysesthesia. In addition, three patients had new-onset seizures during hospitalization, which were controlled with standard antiepileptic therapy. No significant radiation-related adverse events, such as CRN or wound-related complications, were recorded (**Table 3**).

**Table 3:** Immediate and delayed adverse events after IORT.

Adverse events	n (%)
Immediate (≤72h)	
Dysphasia	2 (6.7%)
Muscle weakness	3 (10.0%)
Dysesthesia	2 (6.7%)
Headache	10 (33.3%)
Hydrocephalus	0 (0.0%)
Cerebrospinal fluid leakage	0 (0.0%)
Meningitis	0 (0.0%)
Seizure	3 (10.0%)
Postoperative hemorrhage	0 (0.0%)
Cerebral radiation necrosis	0 (0.0%)
Delayed (>72h)	
Wound infection	0 (0.0%)
Scalp hydrops	0 (0.0%)
Wound dehiscence	0 (0%)

### The effect of post-operative radiotherapy and chemotherapy on survival

Despite the formulation of a personalized treatment plan after IORT formulated, we were surprised to find that half of the patients did not receive radiotherapy or chemotherapy according to the Stupp protocol during follow-up (**Table 2**). Only seven patients received combined external beam radiotherapy (EBRT) and temozolomide-based chemotherapy, while the remaining patients either received radiotherapy or chemotherapy alone.

**Figure 1:**
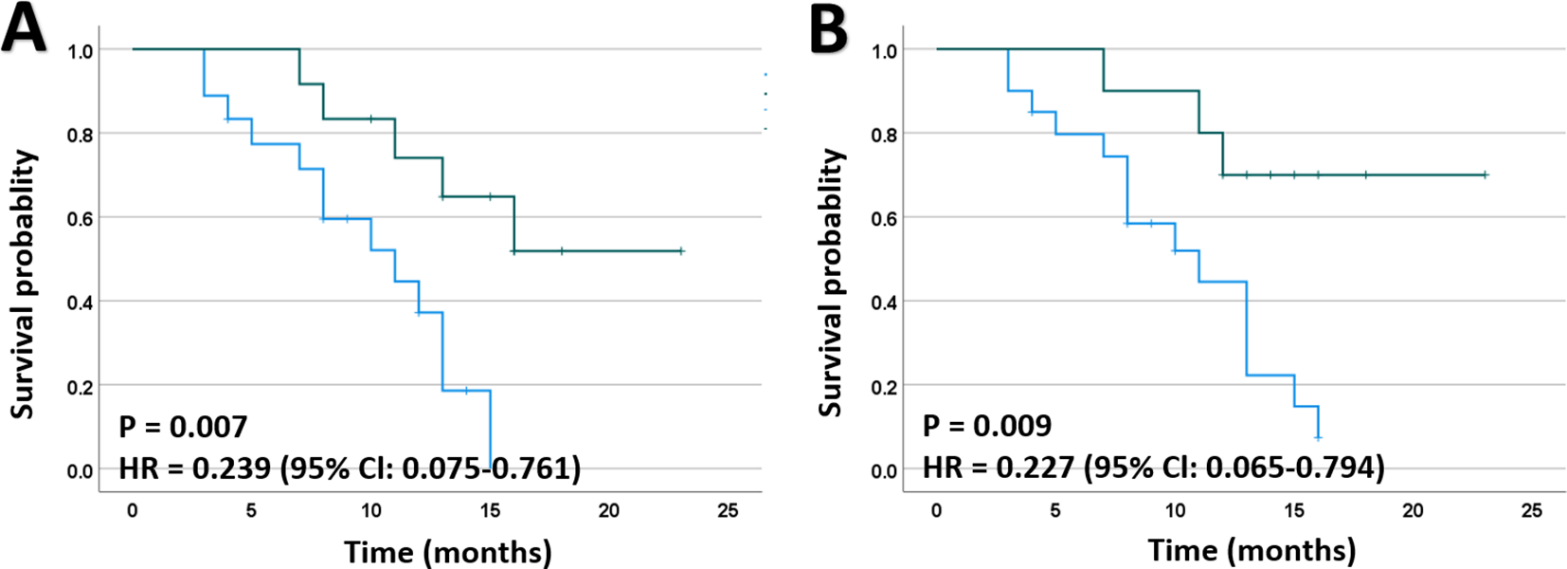
Kaplan-Meier plots for death of patients with HGGs. **(A)** Mortality was significantly lower in patients receiving EBRT after IORT than those who did not. **(B)** Mortality was significantly lower in patients receiving chemotherapy after IORT than those who did not. EBRT, external beam radiotherapy; HR, hazard ratio.

To investigate the effect of post-operative radiotherapy and chemotherapy on survival, we divided all enrolled patients into two groups: those who received post-operative radiotherapy or chemotherapy and those who did not, and then compared their OS between the two groups. As shown in **Figure 1A**, the combination of postoperative radiotherapy and IORT prolonged the OS compared to IORT alone. Similar results were obtained for postoperative chemotherapy (**Figure 1B**).

To further validate the potential impact of post-operative radiotherapy on prognosis, Cox regression analyses were conducted to account for its potential effects on OS. In the initial univariate Cox regression analyses, postoperative EBRT (HR, 0.239; 95% CI, 0.075-0.761; P = 0.007) and chemotherapy (HR, 0.227; 95% CI, 0.065-0.794; P = 0.009) were identified as significant risk factors for death among the patients. Subsequently, a multivariate model including age, Ki-67 labeling index, EBRT, and chemotherapy was established using a forward stepwise approach, which indicated that postoperative EBRT was an independent risk factor for favorable clinical outcomes in HGGs (**Table 4**).

**Table 4:** Cox regression analyses of risk factors for death for patients with HGGs receiving IORT.

Variable	Univariable analysis		Multivariable analysis	
	HR (95% CI)	P value	HR (95% CI)	P value
Age, y	1.025 (0.991-1.059)	0.155	–	–
Male	1.133 (0.443-2.902)	0.794		
Laterality		0.477		
Right-sided	Reference			
Left-sided	0.709 (0.274-1.831)	0.477		
Tumor volume, cm^3^	2.500 (0.801-7.806)	0.115		
Recurrent tumor	1.663 (0.471-5.873)	0.430		
Functional area tumor	1.340 (0.472-3.803)	0.583	–	–
Preoperative KPS, %	1.004 (0.942-1.070)	0.908		
Extent of resection				
Gross total resection	Reference			
Subtotal resection	1.580 (0.452-5.519)	0.473		
Pathology				
Astrocytoma	Reference			
Glioblastoma	1.095 (0.248-4.834)	0.905		
Oligoastrocytoma	5.300 (0.613-45.847)	0.130		
WHO grade				
III	Reference			
III-IV	0.750 (0.229-2.459)	0.635		
IV	0.750 (0.250-2.251)	0.608		
Ki-67, %	1.106 (0.994-1.039)	0.164	–	–
EBRT	0.239 (0.075-0.761)	0.015	0.239 (0.075-0.761)	0.015
Chemotherapy	0.227 (0.065-0.794)	0.020	–	–

The multivariable model contains age, Male, laterality, Tumor volume, Recurrent tumor, Functional area tumor, Preoperative KPS, Extent of resection, Pathology, WHO grade, Ki-67 index, EBRT, and chemotherapy.

EBRT, external irradiation radiotherapy; HR, hazard ratio; KPS, Karnofsky performance scale.

### Illustrative case

**Figure 2:**
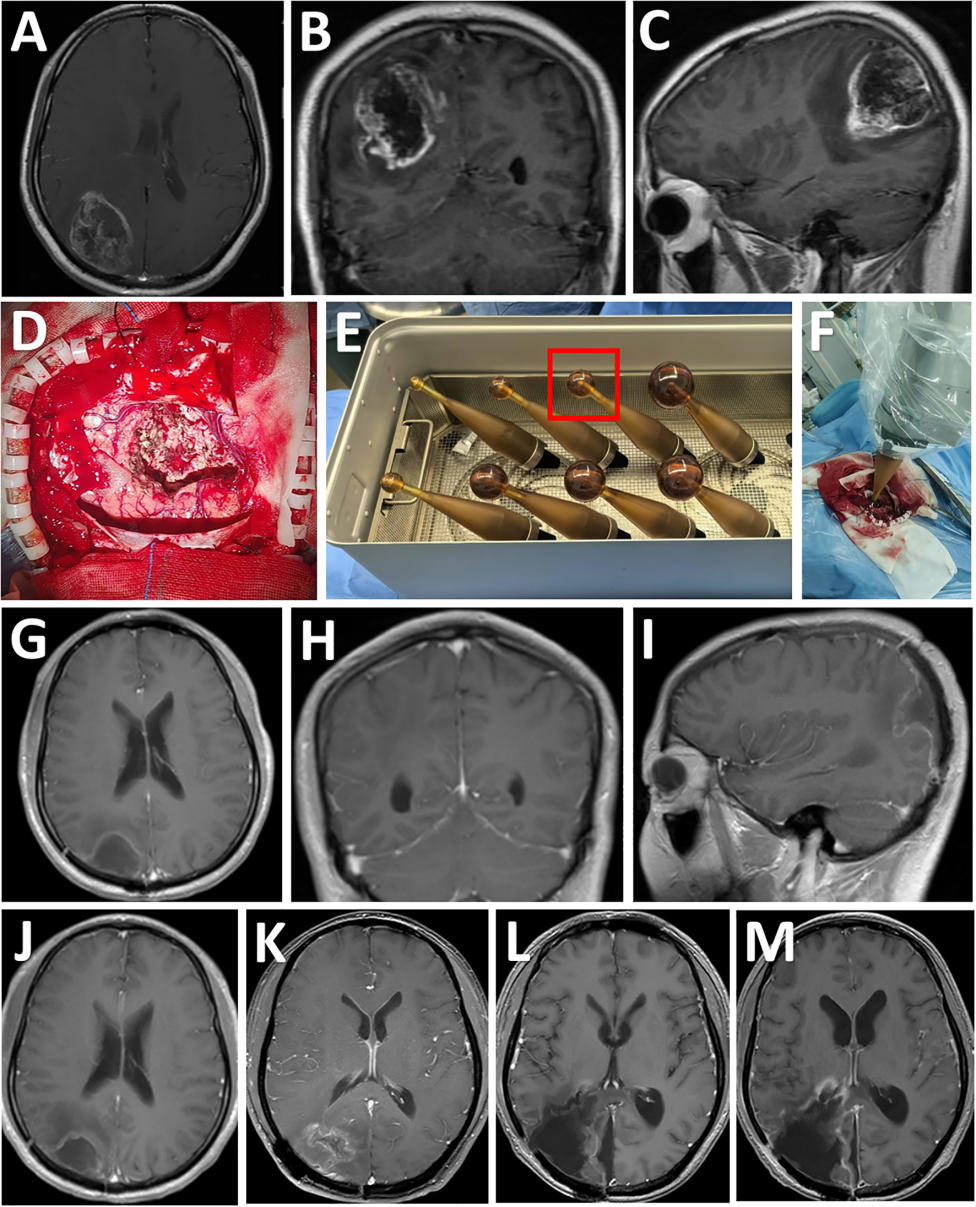
Consecutive MRI follow-up examinations of a patient after IORT. **(A-C)** Preoperative MRI images showing a T1-enhancing lesion diagnosed as Grade 4 astrocytoma after resection in the right parietooccipital lobe (axial, coronal, and sagittal views, tumor size indicated). **(D)** Exposure of the tumor cavity after gross total resection. **(E)** According to the size of resection cavity, a 3.0-cm diameter spherical applicator was selected from a series of available applicators with diameters ranging from 1.5 to 5.0 cm for IORT. **(F)** The applicator was inserted into the residual cavity of the tumor. **(G-I)** Postoperative post-contrast MRI scans performed three months after surgery showing total removal of the lesion. **(J)** Post-contrast MRI scans performed six months after surgery showing no disease progression. **(K)** Post-contrast MRI scans performed eighteen months after surgery showing tumor recurrence. **(L)** Post-contrast MRI scans showing gross total resection of the tumor one month after re-resection. **(M)** Post-contrast MRI scans showing local re-recurrence two months after re-resection.

A 32-year-old male (Patient 2) presented with sudden headache and loss of vision lasting three days. His KPS was 90%. MRI showed a contrast enhanced lesion with intratumoral hemorrhage in the right parietooccipital lobe (**Figures 2A-C**), which was diagnosed as Grade IV astrocytoma after histopathological analysis. Complete tumor resection was performed under the guidance of neuronavigation and sodium fluorescein, followed by low-dose X-rays (12 Gy) based IORT via the INTRABEAM system. Using the 3-cm applicator sphere, the irradiation lasted 15 minutes (**Figures 2D-F**). After surgery, the patient exhibited no new-onset sensorimotor deficits, with a postoperative KPS score of 90%. The patient received standard radio-chemotherapy according to the Stupp protocol after surgery. Postoperative MR imaging confirmed macroscopic complete resection (**Figures 2G-I**). Tumor recurrence was found eighteen months after surgery (**Figures 2J, K**), leading to re-resection. Unfortunately, tumor recurrence was observed again two months after re-resection (**Figures 2L, M**), and the patient died eight months after re-resection.

## Discussion

The rationale for IORT in neuro-oncology is based on its anatomical precision, which allows for the direct application of radiation to the resection cavity, where most recurrences occur. Additionally, IORT offers a temporal advantage of delaying recurrence between surgery and adjuvant therapy during which tumor cells may proliferate (15). IORT can be delivered via low-kV X-rays, high-dose electrons, or high-dose rate brachytherapy (6). While photon-based IORT has been used in the domain of neuro-oncology for decades, X-ray-based IORT gained popularity in recent years (16). Several meta-analyses, including early observational studies without control groups, suggest that IORT may be a promising adjuvant treatment of selected patients with HGGs (16–18) and brain metastases (16). Interestingly, emerging evidence from retrospective or prospective observational studies, particularly those using a matched-pair design, indicated that tumor resection followed by X-ray-based IORT improved the survival of patients with recurrent HGGs, without additional complications (2, 19). In addition, this technique has been proven to be a feasible approach following brain metastasis resection, with comparable long-term outcomes as adjuvant stereotactic radiotherapy (20). In this study, we presented our experience with the efficacy and safety of IORT based on X-rays for the treatment of HGGs.

In a dose-escalation trial, Giordano et al. indicated that patients with primary GB could be treated with IORT (20-40 Gy) with acceptable radiotoxicity. In this cohort, five patients (33.3%) developed CRN and the rate of CRN remained relatively stable with increasing dose (12). Accumulating evidence suggests that 25 Gy is the maximum tolerable IORT dose for patients with unirradiated primary brain tumors (21–23), since a single dose of more than 25-30 Gy would increase the risk of vascular injury and tissue necrosis (24). However, for patients with recurrent GB who underwent postoperative radiotherapy before re-resection, the dose of IORT warrants further discussion. The Radiation Therapy Oncology Group dose-escalation trial documented that the maximal dose of radiosurgery in adults with previously irradiated primary or secondary brain tumors should be less than 24 Gy, 18 Gy, and 15 Gy for tumor diameters < 20 mm, 21-30 mm, and 31-40 mm, respectively (25). Recently, Li et al. reported two patients (9.1%) with recurrent GB who received IORT (30-40 Gy) developed CRN, and one died due to refractory cerebral edema. They postulated that the dose of radiotherapy may play a key role in acute cerebral edema (19). On the contrary, no severe complication was observed in a cohort of 17 recurrent GB patients treated with IORT based on low-dose X-rays (10-12 Gy) (2). For the sake of safety, we chose low-dose IORT (10-12 Gy), as adopted in previous studies (13, 14), in this cohort including patients with primary or recurrent HGGs. Pathological observations have shown that IORT causes a toxic infiltration depth of 2-5 mm at a dose of 10-14 Gy in malignant brain tumors (11, 14, 26). This dose shows limited damage to vessel tissues and white matter fiber bundles (2), and the incidence of CRN induced by IORT is less than 5% using X-ray doses ranging from 10-20 Gy (11, 27). In this cohort, no patient who received IORT had severe complications, suggesting the safety of this dose.

In 2021, Ylanan et al. performed a systematic review of the oncologic outcomes of IORT for GB in 123 patients, and found that the median OS was 13-14.2 months for electron-based studies and 13.8-18 months for photon-based studies (28). Recently, Palavani et al. reported that the 12-month survival rate was 74% for 436 patients with HGGs receiving IORT, based on a meta-analysis that included 16 studies (18). In this study, the median OS was 10.7 months, and the 1-year survival rate was 46.7%, which were inferior to those reported in previous meta-analyses and recent observational studies by Li et al. and Bao et al. (2, 19). We believe this discrepancy in outcomes may be owing to the heterogeneity in study design, patient characteristics, and treatment procedures. First, nearly half of the patients in this cohort had gliomas involving functional areas, and five patients received only subtotal resection. Residual tumor in these cases may lead to early cancer recurrence and poorer outcomes. Second, a low dose of IORT was adopted in this study, and whether the efficacy of IORT strengthens in a dose-escalation way remains to be determined in future research. Third, despite individualized treatment plans determined after surgery and IORT, nearly half of the patients did not receive adjuvant radiotherapy or chemotherapy, which may impact the prognosis. We further divided all enrolled patients into those who received postoperative radiotherapy or chemotherapy and those who did not after IORT, and found that the former group had a longer OS. For example, the selected case (patient number 2) received standard postoperative chemotherapy and radiotherapy after IORT, and this patient’s survival time (26 months) was longer than the median survival time (11 months). These data suggest that comprehensive treatments are necessary to improve clinical outcomes for HGGs after IORT.

In this study, we categorized complications into immediate and delayed events. Regarding immediate complications, two patients experienced dysphasia, three exhibited muscle weakness, two had dysesthesia, ten experienced headaches, and three had seizures postoperatively. We believe these complications may be related to surgery or ischemia, and most patients recovered following appropriate medical treatment. Currently, there is no evidence suggesting that radiation encephalopathy was caused by IORT. As for delayed complications, none were observed in this cohort, which may be attributed to the relatively low dose of radiation used in this study. This study included four patients with glioblastoma, whose average survival time was 9.5 months; 25 patients with astrocytoma, whose average survival time was 10.4 months; and one patient with oligoastrocytoma, who survived for 7 months. For WHO grade III tumors, there were six patients with an average survival time of 12.7 months. For patients with WHO grade III-IV tumors, the survival time averaged 9.8 months, and for grade IV tumors, the average survival time was 9.9 months. Due to the limited sample size, it is difficult to draw definitive conclusions regarding the direct relationship between survival time and pathological type or WHO grading. Prior to the surgical procedure, the patients had a median KPS score of 80 (IQR: 75-80). Six months after IORT, the patients had a median KPS score of 70 (IQR: 50-75), representing a 12.5% decline in their functional status. Several studies have shown the superiority of IORT in terms of local control, with similar overall survival outcomes compared to conventional radiotherapy for gliomas ^2,12.19^. A comparison with other clinical trial data from this study is shown in **Table 5**.

**Table 5:** Comparison with previous study on IORT.

Study	Our study	Bao 2023	Li 2023	Giordano 2019
N	30	17	21	15
Age	mean 62	mean 44	mean 52	mean 62
Sex (M/F)	16/14	8/9	7/14	8/7
Tumor	25 A, 4 GB, 1 OA	17 GB	17 GB, 4 Gliomas of WHO III	15 GB
Karnofsky status pre-op	mean 80%	NA	mean 70%	mean 80%
Dose IROT	10-12Gy	10-12Gy	30-40Gy	20-40Gy
OS (month)	mean 11.0	mean 12.8	mean 13.5	mean 17.8

M, Male; F, Female; GB, glioblastoma; A, astrocytoma; OA, oligoastrocytoma; Gy, Gray.

This study has several limitations: First, it was a single-center study with a small sample size and non-random patient selection, which may result in a sample that does not fully represent the target population. This introduces significant biases that can distort the study conclusions and limit its real-world applicability. Potential solutions include stratified sampling, statistical weighting, and transparent reporting of selection criteria to clarify the boundaries of generalizability. Second, the available data failed to deal with the heterogeneity of HGGs, such as pathological and immunogenetic features, and further studies with a larger cohort should incorporate these additional factors. Third, the efficacy of different doses of IORT in HGG therapy is urgently necessitated. Prospective multicenter trials (NCT02685605) are currently underway to validate dose-escalation protocols. Future studies should incorporate molecular profiling (e.g., IDH status) or neuroimaging clustering analyses (29) to enable personalized IORT strategies. Despite these shortcomings, we believe these data may provide guidance for neurosurgeons to gain a better understanding of the efficacy and safety of low-dose IORT in the treatment of HGGs.

## Conclusion

This study confirms the safety of low-dose (10 to 12 Gy) X-ray based IORT in the treatment of HGGs. However, further studies through prospective, large-scale, randomized controlled trials, are necessitated to verify the efficacy of IORT with varying doses as a therapeutic option for this disease. Even with the use of IORT and tumor resection, postoperative radiotherapy is necessary to further improve the clinical outcomes of these patients. This clinical study provides additional clinical data on IORT for HGGs, which may represent a promising therapeutic approach for managing these tumors. Based on previous results and the treatment of HGGs, an important consideration is how to manage tumor boundaries effectively. Future treatment protocols for HGGs should not only include intraoperative radiation therapy but also integrate techniques such as intraoperative fluorescence, pathology, and navigation to minimize the risk of tumor recurrence at the original site.

## Data Availability

The original contributions presented in the study are included in the article/supplementary material. Further inquiries can be directed to the corresponding author/s.
